# Gut Microbiota Is Associated with Onset and Severity of Type 1 Diabetes in Nonobese Diabetic Mice Treated with Anti–PD-1

**DOI:** 10.4049/immunohorizons.2300103

**Published:** 2023-12-26

**Authors:** Shriram Patel, Eugenia Becker, Corinne Ploix, Guido Steiner, Petar Scepanovic, Matthias Fueth, Maria Cristina de Vera Mudry, Anne Eichinger-Chapelon, Estelle Marrer-Berger, Marcus J. Claesson

**Affiliations:** * School of Microbiology and APC Microbiome Ireland, University College Cork, Cork, Ireland; † SeqBiome Ltd, Cork, Ireland; ‡ Pharmaceutical Sciences, Roche Innovation Center Basel, Pharma Research & Early Development, Hoffmann-La Roche, Basel, Switzerland

## Abstract

Our bodies are home to individual-specific microbial ecosystems that have recently been found to be modified by cancer immunotherapies. The interaction between the gut microbiome and islet autoimmunity leading to type I diabetes (T1D) is well described and highlights the microbiome contribution during the onset and T1D development in animals and humans. As cancer immunotherapies induce gut microbiome perturbations and immune-mediated adverse events in susceptible patients, we hypothesized that NOD mice can be used as a predictive tool to investigate the effects of anti–PD-1 treatment on the onset and severity of T1D, and how microbiota influences immunopathology. In this longitudinal study, we showed that anti–PD-1 accelerated T1D onset, increased glutamic acid decarboxylase–reactive T cell frequency in spleen, and precipitated destruction of β cells, triggering high glucose levels and pancreatic islet reduction. Anti–PD-1 treatment also resulted in temporal microbiota changes and lower diversity characteristic of T1D. Finally, we identified known insulin-resistance regulating bacteria that were negatively correlated with glucose levels, indicating that anti–PD-1 treatment impacts the early gut microbiota composition. Moreover, an increase of mucin-degrading *Akkermansia muciniphila* points to alterations of barrier function and immune system activation. These results highlight the ability of microbiota to readily respond to therapy-triggered pathophysiological changes as rescuers (*Bacteroides acidifaciens* and *Parabacteroides goldsteinii*) or potential exacerbators (*A. muciniphila*). Microbiome-modulating interventions may thus be promising mitigation strategies for immunotherapies with high risk of immune-mediated adverse events.

## Introduction

As the use of cancer immunotherapy (CIT) has become common, immune-mediated adverse events (imAEs) such as chronic inflammation, autoimmunity, and hypersensitivity reactions have emerged as its Achilles’ heel. Checkpoint receptors, such as PD-1 and CTLA-4, are key players in maintaining self-tolerance by controlling the duration and amplitude of physiological immune responses and thus limiting collateral damage and preventing autoimmunity. Blocking these tolerance-inducing pathways is consequently linked to the occurrence of associated imAEs, which may lead to CIT-induced autoimmune diseases (1–4). As new CIT therapies are on the forefront, it is critical to understand the contribution of different factors to the onset and severity of imAEs in an attempt to optimize the therapeutic index for each patient and avoid premature cessation of therapies. The NOD mouse has proven to be a valuable model for studying autoimmune diabetes, which occurs spontaneously, includes multiple genetic mutations (polygenic), and involves innate and acquired immunity. The pathophysiological mechanisms leading to type 1 diabetes (T1D) in NOD mice are well characterized and offer robust translation to human disease (5). However, insulitis is not uniformly progressing to disease, and the mechanisms regulating the initiation and severity of insulitis to overt diabetes are less understood. It is believed that environmental factors (e.g., diet, lifestyle, microbiota) and genetics plays a crucial role here. As ∼70–80% of immune cells are located in the gut, it is crucial to consider that trillions of microbes have the potential to prime immune cells via multiple pattern recognition patterns (PRRs), such as TLRs or nucleotide-binding and oligomerization domain-like receptors (NLRs) (6).

On the one hand, it has been suggested that the microbiota modulates T1D onset and course in NOD mice, but that it is not the cause of disease, as germ-free NOD mice show similar incidences of insulitis compared with mice kept under specific pathogen-free (SPF) conditions (7). Furthermore, MyD88^−/−^ NOD mice under SPF conditions were protected from T1D (8); however, this was not seen under germ-free conditions. Additionally, TLR2^−/−^ NOD mice were shown to be resistant (9) and TLR9^−/−^ NOD mice to be protective against T1D (10), whereas TLR4^−/−^ NOD mice induced accelerated autoimmune diabetes under SPF conditions (11). On the other hand, PD-1 and CTLA-4 treatments seem to result in early onset of diabetes in NOD mice, which is in line with the mode of action of these molecules and is also observed in humans clinically (12, 13). The effects of checkpoint inhibitors (PD-1/PD-L1 and CTLA-4) on the microbiota have also been described in multiple reports and cancer mouse models (14–16).

In this study, we applied the protocol from Ansari et al. (17), complemented with gut microbiota evaluation to further investigate its influence on the incidence, onset, and severity of diabetes developed during early life spans by NOD mice treated with a murine anti–PD-1 Ab. We found that anti–PD-1 treatment and T1D altered the microbiota composition and diversity and identified insulin-regulating bacteria that may have reduced glucose levels over time. To our knowledge, this is the first time a study investigated how anti–PD-1 treatment is shaping the microbiome longitudinally in an autoimmune mouse model. In this study, we evaluated this predictive model for its applicability to future CITs in determining potential safety risks based on early safety biomarkers within the microbiota and host immune responses.

## Materials and Methods

### Animals

NOD (NOD/ShiLtJ), BALB/c (BALB/cAnNCrl), and NOD-SCID (NOD.CB17-Prkdc^scid^/NCrCrl) female mice were purchased from Charles River Laboratories (Sulzfeld, Germany). Animals were aged ∼5 wk at the start of dosing. They were kept in an air-conditioned room under periodic bacteriologic control at a temperature of 22 ± 2°C, with 40–80% humidity and a 12-h light/12-h dark cycle and background music coordinated with light hours. Mice were housed in groups of two or three in environmentally enriched Makrolon type III boxes with autoclaved sawdust bedding. Additional filter top and HEPA filter changing stations were used for NOD-SCID mice. A pelleted standard rodent diet and tap water were supplied ad libitum. Animals were randomly assigned to dose groups based on body weight using the data collection software Provantis v9.4 (Instem Life Sciences, Stone, U.K.). This study was not performed under good laboratory practice. The animals were kept in a facility accredited by the American Association for Accreditation of Laboratory Animal Care International and treated in accordance with the guidelines of the Swiss Animal Welfare Act. All procedures were in accordance with the respective Swiss regulations and approved by the Cantonal Ethical Committee for Animal Research.

### Study design

A 2-wk toxicity study was conducted in 55 mice. Mice were divided into five groups. Three groups of 15 NOD mice each were either 1) untreated (naive) to evaluate the onset and worsening of T1D in the model itself as reference, 2) treated with an unspecific isotype IgG as vehicle (InVivoMAb rat IgG2a isotype control, anti-trinitrophenol, Bio X Cell, NH, USA), or 3) treated with the InVivoMAb anti-mouse PD-1 (CD279) Ab from Bio X Cell (Lebanon, NH). Five BALB/c were added to assess the effect on the microbiome in the same room with the same food and the same age compared with genetically modified animals, and five NOD-SCID mice were allocated to the fifth group as these immunosuppressed mice do not develop T1D. The test items were administered i.v. in the tail vein at 500 µg/administration on day 1 and 250 µg/administration every second/other day for five administrations. The dosing period was followed by a treatment-free observation phase of 3–4 wk to assess the onset and progression of diabetes. Both dose and dose frequency were selected on the basis of a combination of in vitro (T cell activation assay and cytokine release) and in vivo data indicating the duration of response and exposure required for efficacy in mice. Clinical signs, body weight development, and food consumption were monitored throughout the study. Blood was taken before treatment, during treatment, and after the treatment phase. Lymph nodes, spleen, and pancreas were taken on days 12, 17, and 38. Fecal samples were collected before treatment, during treatment, and three times after treatment from all groups of the study.

### Monitoring diabetes via glucose measurement

To monitor the onset of insulin-dependent diabetes mellitus, daily blood sampling for glucose measurement was performed by puncture of the tail tip without sedation (only groups 1, 2, and 3). A sample volume of 0.6 μl is required for analysis with Accu-Chek Advantage glucometers (Roche Diagnostics, Evaluation Report: Accu-Chek Aviva Test Strips 2013). Onset of type 1 diabetes was defined as a random blood glucose reading of 250 mg/dl (14 mmol/L) or greater for three consecutive occasions. Blood glucose levels were measured once prior to start of the study, daily for the first 11 d during treatment, and two to three times per week during the treatment-free posttreatment observation period.

A terminal nonfasted blood sample for laboratory investigations was drawn sublingually under light isoflurane anesthesia from all animals shortly before necropsy and without overnight fasting. Animals were sacrificed immediately after blood collection without waking up. Approximately 50 μl of blood sampled into EDTA tubes was used for measuring hematologic parameters. Serum samples from at least 500 μl of blood were used for clinical chemistry assays and quantification of electrolytes.

### Bioanalytics

Blood samples were obtained from group 3 animals at the end of the administration phase (day 11, three animals [10 min] before the last dose and three animals [10 min] after last dose) as follows to confirm exposure. Animals were not fasted before bleeding. Approximately 50 μl of blood was collected without anesthesia from the tail vein in K3-EDTA tubes. After centrifugation at ∼1500 × *g*, for ∼10 min at 4°C, the resulting plasma was used in a sandwich ELISA quantifying against anti-rat PD-1–IgG. Owing to limitations in sample numbers, no pharmacokinetic evaluation were performed and only exposure confirmation for the provided sample time points are reported in this study.

### Immunopathology: tetramer staining and ELISPOT

ELISPOT for glutamic acid decarboxylase (GAD)–specific T cells and tetramer FACS of insulin-specific splenocytes were performed, aiming at quantifying autoreactive T cells upon anti–PD-1 treatment. Spleen and draining lymph nodes from 10 animals per group in groups 1, 2, and 3 were collected in individual tubes on days 17 and 24 and the five animals in group 5 on study termination.

As for the tetramer staining, single cell suspensions were obtained by mincing the tissue in a petri dish and mashing it on top of a cell strainer. Cell suspensions (10^7^ cells) were stained with islet-specific glucose-6-phosphate catalytic subunit-related protein (IGRP)206-214 (H-2K^d^ IGRP tetramer-VYLKTNVFL-PE, MBL International, no. TB-M552-1) for 30 min at room temperature, washed, and incubated with anti-PE MicroBeads (Miltenyi Biotec, no. 130-048-801) for 15 min at 4°C. Magnetic separation was performed using an autoMACS Pro system (Miltenyi Biotec). Tetramer-bound cells were quantified by FACS using CD8 allophycocyanin (Bio-Rad clone KT15 IgG 2a, no. MCA609APC), CD3 BV421 (BioLegend clone 145-2C11, no. 100336), F4/80 AF488 (BioLegend clone BM8, no. 123120), B220 AF488 (BioLegend clone Ra-3-6B2, no. 103225), CD11b AF488 (BioLegend clone M1/70, no. 101217), CD11c AF488 (BioLegend clone N418, no. 117311), and BD Horizon fixable viability stain 510 (excitation maximum of 408 nm, fluorescence emission maximum of 512 nm; Violet V500, BV510, 0.1 mg; BD Biosciences, no. 564406). From the live cell population, CD11c^−^CD11b^−^B220^−^F4/80^−^CD3^+^ cells were identified as the T cell population for analysis. The CD8^+^ T cells were then gated, and the IGRP tetramer–positive population was quantified on a FACSFortessa (BD Biosciences) using FlowJo software (Tree Star).

ELISPOT analysis was applied to quantify the frequencies of IFN-γ–producing GAD-reactive T cells and assess the state of activation of T cells in mice receiving anti–PD-1 or vehicle. In brief, spleen and draining lymph nodes from treated and control mice were processed into single-cell suspensions. ELISPOT plates (Mabtech, no. 3321-4APW-2) were blocked with PBS-FCS 10% for 1 h at room temperature and then washed with PBS. Cells were plated (5.10^4^/well) in complete RPMI 1640 medium containing 10% FCS (Sigma-Aldrich), 2 mM l-glutamine, 100 U/ml penicillin/streptomycin (BioWhittaker), and 50 mM 2-ME (Sigma-Aldrich). Control wells contained unstimulated cells or medium alone, whereas GAD Ag (10 μg/ml, Abcam, no. 206646) or Con A (5 μg/ml, Sigma-Aldrich) was added to stimulated wells. After a 48-h incubation at 37°C and 5% CO_2_, the plates were washed five times. Biotinylated detection mAbs (R4-6A2–biotin, 1 μg/ml) was added and the plates are left for an additional 2-h incubation at room temperature. Streptavidin–alkaline phosphate (1:1000) was then added for 1 h at room temperature followed by substrate solution (BCIP/NBT-plus) until distinct spots emerged (10–30 min). Color development was stopped by washing extensively in tap water and the plate was let to dry. The resulting spots were counted on a computer-assisted ELISPOT image analyzer (Cellular Technology).

### Light sheet microscopy

#### Tissue clearing

One naive mouse (blood glucose 5.8 mmol/l/104 mg/dl) and one anti–PD-1 mouse surrogate–treated mouse (blood glucose 30.7 mmol/l/>286.5 mg/dl) designated for light sheet microscopy were sacrificed on day 37 of the study. The entire pancreas (splenic and duodenal parts labeled) of both mice was dissected at necropsy and fixed for 24 h in 4% PFA at 4°C. The pancreas was cleared using the CUBIC (clear, unobstructed brain/body imaging cocktails and computational analysis) method. Briefly, the pancreas was incubated in CUBIC-1 solution on a shaker (90 rpm) at 37°C. CUBIC-1 solution was changed after ∼12 h, 1–4 d, until the pancreas was completely cleared. The pancreas was then washed in PBS three to five times and placed in PBS overnight on a shaker (90 rpm) at 37°C. The pancreas was incubated in CUBIC-2 solution, on a shaker (90 rpm) at 37°C for 2 d, with daily changes of CUBIC-2 solution. The pancreas was then kept in CUBIC-2 solution at room temperature in the dark prior to immunostaining.

#### Immunohistochemistry

Cleared pancreas was immunostained for chromogranin A. All steps were performed in closed, fully filled tubes to prevent oxidation. The pancreas was incubated in permeabilization solution at 37°C for a maximum of 2 d, followed by incubation in blocking solution at 37°C for a maximum of 2 d. The pancreas was then incubated in primary Ab (1:100 polyclonal rabbit anti–chromogranin A [Novus Biologicals, no. NB120-15160], 1:250 in DAPI [1/100] in PTwH [PBS/0.2% Tween 20 with 10 mg/ml heparin] [5%]/DMSO [3%] goat serum) in PTwH (5%)/DMSO (3%) donkey serum at 37°C for 4 d, followed by washing in PTwH four to five times until the next day. The pancreas was then incubated in secondary Ab (Alexa Fluor 488 donkey anti-rabbit [Invitrogen, no. A-21206] in PTwH/3% donkey serum) at 37°C for 4 d, followed by washing in PTwH four to five times until the next day. The pancreas was imaged in fresh CUBIC-2 solution using LaVision ultramicroscope, and acquired images were further processed with arivis software.

### DNA extraction, library preparation, and 16S rRNA gene sequencing

DNA was extracted from mouse fecal pellets with the QIAamp Fast DNA stool mini kit (51604) using a modified extraction method. Fecal pellets were weighed (average weight 0.058 ± [SD] 0.027 g) and added to a sterile 2-ml microcentrifuge tube containing one 3.5-mm glass bead, 0.1 ml of 1.0-mm zirconia/silica beads, and 0.1 ml of 0.1-mm glass beads (BioSpec, Bartlesville, OK). Then, 1 ml of Inhibitex buffer was added to the fecal samples, which were disrupted by bead beating in a Mini-Beadbeater-16 (BioSpec) at maximum speed (3450 strokes/min). Samples were disrupted for 1 min, followed by incubation on ice for 1 min, disrupted a second time for 30 s, and placed back on ice before incubation at 95°C for 7 min. Samples were vortexed for 15 s and centrifuged at 13,300 rpm for 1 min to pellet the stool particles, after which 400 µl of the supernatant was added to a sterile 1.5-ml microcentrifuge tube containing 20 µl of proteinase K. Then, 400 µl of buffer AL was then added to the tube and the samples were vortexed for 15 s before incubation at 70°C for 10 min, after which 400 µl of ethanol was added to the lysate and thoroughly mixed by gently pipetting the mixture. Lysate (650 µl) was added to the QIAamp spin column and centrifuged for 1 min at 13,300 rpm and the filtrate was discarded. The remainder of the lysate was added to the QIAamp spin column, which was centrifuged again under the same conditions before continuing with the DNA extraction as per the kit manufacturer’s instructions until the DNA elution step. The DNA was eluted by applying 50 µl of buffer ATE to the QIAamp spin column membrane and incubating at room temperature for 3 min before centrifugation to elute the DNA (1 min at 13,300 rpm). This eluate was then applied to the QIAamp spin column membrane a second time and incubated and centrifuged as before to finally elute the DNA.

DNA quality and concentration were measured using a NanoDrop 2000 spectrophotometer (Thermo Scientific, Waltham, MA), and subsequently the DNA was stored at −80°C prior to PCR amplification. Library preparation for 16S rRNA gene amplicon sequencing was performed following the Illumina (San Diego, CA) recommendations with some modifications. Briefly, aliquots of 30 ng of extracted genomic DNA were subjected to PCR amplification of the V3–V4 hypervariable region (341 forward, 5′-CCTACGGGNGGCWGCAG-3′ and 805 reverse, 5′-GACTACHVGGGTATCTAATCC-3′) of the 16S rRNA gene in a total PCR reaction volume of 30 µl. The PCR primers were selected from Klindworth et al. (18). Illumina adapters containing overhang nucleotide sequences were added to the gene-specific sequences and used at a concentration of 0.2 µM.

PCR amplification with Phusion HIGH-FIDELITY DNA polymerase (Thermo Scientific) was performed on a SimpliAmp thermal cycler (Applied Biosystems) under the following conditions: 98°C for 30 s, followed by 25 cycles of 98°C for 10 s, 55°C for 15 s, 72°C for 20 s, and a final cycle of 72°C for 5 min before cooling to 4°C. Pre- and post-PCR steps were performed in separated and designated areas of the building. The successful generation of the PCR amplicon was verified by observing the PCR product band in 1% agarose gels.

After PCR, amplified products were purified using Agencourt AMPure XP magnetic beads (Beckman Coulter, Brea, CA) and eluted in 52.5 µl of EB buffer (Qiagen). After this initial purification, 5 µl of the purified amplicon–PCR product was amplified in a second PCR to add the Illumina barcode sequences to the 16S gene-specific sequences. This was performed on a 2720 thermal cycler (Applied Biosystems, Foster City, CA) employing Nextera XT v2 index primer kits (Illumina). The PCR conditions were 98°C for 30 s, followed by eight cycles of 98°C for 10 s, 55°C for 15 s, 72°C for 20 s, and a final cycle of 72°C for 5 min before cooling to 4°C. A second purification step with Agencourt AMPure XP magnetic beads was carried out after the Nextera PCR. These 16S V3–V4 rRNA gene amplicons containing the Nextera indexes were finally eluted in 27.5 µl of EB buffer, and the concentration of each sample was measured using a Qubit 3 fluorometer (Invitrogen) employing the Qubit dsDNA HS assay kit. Pooled libraries were created by combining 40 ng of each sample. Negative PCR controls and extraction kit negative controls were processed alongside study samples and were also sequenced. A sample of the final library pool was sequenced at the Teagasc next-generation sequencing facility (Teagasc Moorepark, Fermoy, Ireland) on an Illumina MiSeq generating 2 × 300-bp paired end reads.

### Sequence data processing and analysis

Quality of raw reads were checked using FastQC v0.11.5 (19) followed by quality filtering using TrimGalore v0.6.5 (20) with following parameters: --paired, --nextera, --stringency 7, --quality 20, --length 150. A big data pipeline was used to infer ribosomal sequence variants (RSVs) using DADA2 v1.12 (21) considering following parameters: truncLen=c(261,227), trimLeft=c(17, 21), maxEE=c(1, 1), truncQ=c(2, 2), maxN=0, rm.phix=TRUE. In brief, stringent quality filtering was performed with the filterAndTrim function. In that, reads that mapped to the phiX genome were removed, forward (261 bp) and reverse (227 bp) reads were truncated to specific position, and reads that contained unambiguous base or bases with a quality score <2 were discarded, and the maximum number of expected errors per read was set to 1. Then, DADA2 error correction and chimera removal steps were carried out to infer RSVs. The resulting nonchimeric RSVs were again chimera filtered using reference-based chimera filtering implemented in USEARCH v11 (22). Taxonomy was assigned to nonchimeric sequences in QIIME 2 (23) using the SILVA 138 reference database (24). Additionally, we used SPINGO for species level classification whenever possible (25).

Potential reagent contaminants were identified and removed using a frequency-based algorithm implemented in the decontam package (26). In total, 7285 unique RSVs were identified, of which 215 were found to be potential contaminants and thus removed from further analysis. Samples with <5000 (*n* = 1) reads were excluded from further analysis. Next, data filtering was carried out to include RSVs present in >5% of samples with percent abundance of >0.01. Except in the case of α diversity, this filtered RSV count table was used for all the downstream bioinformatics analyses.

We excluded samples from animal number 359 (from group 3) because we found a pathogen genus *Shigella* that was already present in baseline samples pointing to an early colonization. While this genus did not have a strong impact on diabetes or anti–PD-1 treatment, we excluded this animal from our analyses to eliminate the introduction of a potential bias.

### Imputed metagenomics

To investigate functional potential of the murine microbiota after anti–PD-1 treatment, we inferred abundance of Kyoto Encyclopedia of Genes and Genomes (KEGG) pathways (27) in the samples based on the 16S rRNA RSV count table and representative sequences for each RSV using Piphillin2 (28). Briefly, this tool predicts functional gene content based on metataxonomic data with high accuracy by leveraging the most up-to-date version of the KEGG reference database.

### Statistical analysis

All statistical analysis and graphical representations were performed in R using CoDaSeq (29), zCompositions (30), vegan (31), ggplot2 (32), and ggpubr (33) packages. To account for the complex compositional nature of the microbiome data, we used the CoDaSeq pipeline. In brief, we imputed zeros utilizing the count zero multiplicative replacement method (cmultRepl, method = “CZM”) implemented in the zCompositions package and applied centered log-ratio (CLR) transformation of the data using the codaSeq.clr function from the CoDaSeq package.

The α diversity was determined using Chao1, the Shannon index, and the inverse Simpson index. Differences in α diversity within/between groups and between termination rounds (in group 3) were determined using a Kruskal–Wallis test. For principal component analysis, we performed a CLR transformation implemented in the CoDaSeq R package and calculated Euclidean distances, which correspond to Aitchison distances. Permutational multivariate ANOVA (PERMANOVA) (34) was performed on the Aitchison distances with 9999 permutations to test the significance of different clinical variables on mice gut microbiota composition.

To find the association of microbial abundances with different clinical variables, we performed multivariate analysis using multivariate analysis by linear models (MaAsLin2 v1.1.1) in R (35). MaAsLin2 performs boosted, additive general linear models between metadata and microbial abundance. Boosting of metadata and selection of a model were performed per taxon. Microbial abundances were CLR transformed at each taxonomic level to account for the compositional nature of the data. To identify microbial taxa and pathways that are associated with different groups, we carried out 1) between-group analysis considering time point as a fixed effect and animal ID as a random effect, and 2) between-group analyses per individual time point. Similarly, to identify microbial taxa and pathways that are associated with different termination rounds in group 3, we carried out 1) between–termination round analysis considering time point as a fixed effect and animal ID as a random effect, and 2) between–termination round analysis per individual time point. Multiple testing correction was carried out via false discovery rate (FDR) estimation, and associations were considered significant below a FDR value threshold of 0.10.

Next, we evaluated groupwise correlation differences between glucose level and CLR transformed abundance of microbiota using repeated measure correlation (rmcorr) analysis (36). By using this approach, we accounted for compositional effects via CLR and repeated measurements via rmcorr (CLR + rmcorr). Multiple testing correction was carried out via the Benjamini–Hochberg method, and correlation was considered statistically significant below an adjusted *p* value cutoff (FDR < 0.10) of 0.10.

### Data availability

16S rRNA amplicon sequences presented in this article have been submitted to the NCBI BioProject (https://www.ncbi.nlm.nih.gov/bioproject/) under accession no. PRJNA891035.

## Results

**FIGURE 1.**
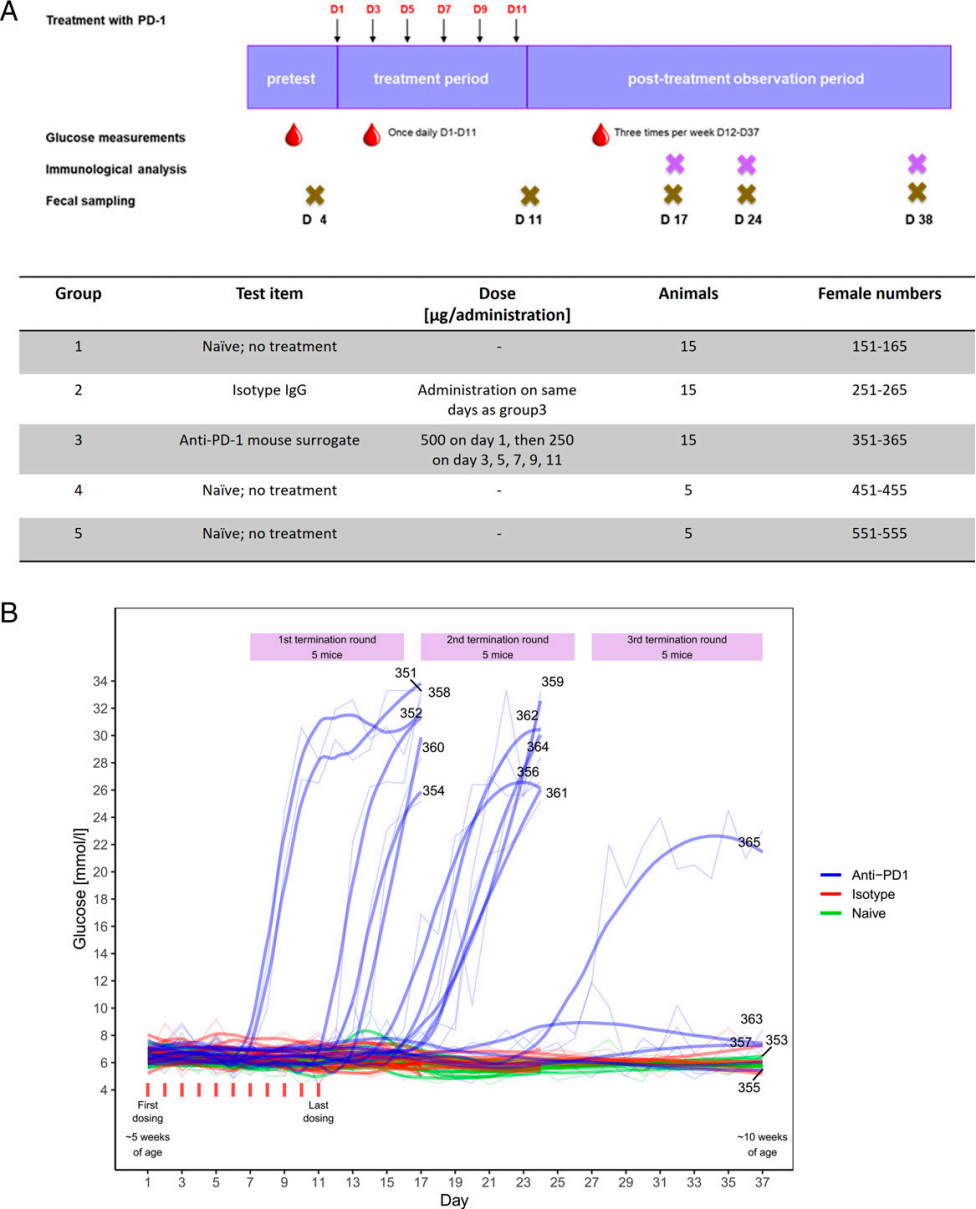
Experimental study design and measurement of changes in glucose levels. (**A**) Stool collection was performed longitudinally from all five groups: pretreatment, directly after treatment cessation, at first termination round (day 17), at second termination round (day 24), and at end of the study (day 38). All mice were 5 wk old at the start of treatment and were aged ∼10 wk when they were terminated. Groups 1–3 are NOD/ShiLtJ (*n* = 15 per group); controls (group 4; *n* = 5) were BALB/c, NOD-SCID (group 5; *n* = 5). Glucose measurement was performed pretreatment, daily during treatment, and three times per week in the posttreatment observation period. Immunological analysis was done at three occasions based on high glucose levels in the posttreatment phase. (**B**) Changes in glucose levels were measured once daily while on treatment. Only the anti–PD-1–treated group (group 3) showed high glucose levels on three different time points, which resulted in three termination rounds. Control groups (BALB/c and NOD-SCID mice) were terminated at the end of the study on day 38.

To evaluate the NOD model as a potential predictor of CIT-associated risks for autoimmunity, we treated NOD mice with a murine anti–PD-1 as previously described (17) and integrated a longitudinal microbiota analysis as a novel component (Fig. 1A). We included parameters such as glucose levels and IFN-γ–producing GAD-specific splenocytes for monitoring incidence, onset, and severity of autoimmune diabetes at three time points (see *Materials and Method*s for details) in three different mouse strains. We also applied light sheet microscopy to demonstrate the heterogeneity of islets in the pancreas and collected fecal samples to evaluate the changes in the microbiota after anti–PD-1 treatment (group 3) and between different mouse strains (NOD/ShiLtJ, groups 1–3; wild-type BALB/c, group 4; and NOD/SCID, group 5).

### Incidence of insulitis onset and glucose levels

The onset of T1D was diagnosed based on three consecutive elevated glucose levels (>14 mmol/l), after which animals were rapidly terminated for further analysis. Acceleration of T1D onset was observed only in group 3 with a total of 11 out of 15 mice ultimately diabetic (Fig. 1B). For each termination round an equal number of isotype-treated and naive mice were also sacrificed, except on day 17, where only two naive mice were euthanized. The first termination round of animals with high glucose levels occurred on day 17 for one third of the group 3 mice. The second termination round included five mice from group 3 on day 24, and the third termination round occurred on day 38, wherein only one animal (no. 365) exhibited high glucose levels, but not the remaining animals (nos. 355, 357, 353, and 363), which stayed nondiabetic until the end of the study).

### GAD-reactive T cells and IFN-γ production

Detection of self-reactive T cells in lymphoid structures at the vicinity of the pancreas is a hallmark of the autoimmune process taking place in the prediabetic stages of T1D. Therefore, to better appreciate differences between treatment groups, we quantified the frequencies of GAD-reactive T cells and insulin-specific CD8^+^ T cells in the spleen (and pancreas-draining lymph nodes) of termination round 1 and 2 animals using IFN-γ ELISPOT and IGRP tetramer staining, respectively (Supplemental Fig. 1). Splenocytes from all three animal groups showed comparable ability to produce high IFN-γ in response to Con A, a mitogenic stimulus. In contrast, unstimulated cells did not exhibit any detectable signal. Interestingly, anti–PD-1 treatment (group 3) significantly increased GAD-specific T cell numbers in the spleen and pancreas-draining lymph nodes of animals relative to group 1 and group 2 mice (*p* < 0.05, Supplemental Fig. 1A). Within the anti–PD-1–treated animal group weaker responses were detected after GAD65 than after Con A stimulation, likely reflecting the lower frequency of the responding population due to the Ag specificity restriction in comparison with lectin-reacting cells. Using the tetramer technology to follow another autoantigenic population, insulin-specific CD8 T cells staining from spleen showed no significant differences between group 1 and group 3 (Supplemental Fig. 1B).

### Three-dimensional pancreas reconstruction

After performing standard histological evaluation by H&E staining, we observed immune cell infiltration in all three NOD groups by day 38. However, we did not observe marked differences between the three NOD groups and sought whether infiltration of islets could be further evaluated by light sheet microscopy. This technology depicts a three-dimensional reconstruction of the pancreas to allow a better understanding of the distribution and volume of the islets and overcome biases that might be introduced by performing histology on a slide-by-slide basis. Supplemental Fig. 2 illustrates islet staining of the pancreas of one animal in group 1 and group 3, respectively. Due to the low animal number, no statistics or further conclusions were possible; however, during the processing we observed that tissue consistency differed between the control (Supplemental Fig. 2A) and anti–PD-1–treated (Supplemental Fig. 2B) animal, with anti–PD-1–treated pancreas being soft and fragile compared with solid of the control mouse. In addition, carefully looking at the islet distribution and staining intensity pointed toward reduced islet number and staining intensity in the anti–PD-1–treated animal. This assessment is for illustration only, and further analysis with a larger sample size may be required.

### Impact of microbiota on insulitis onset and progression

We longitudinally investigated the bacterial composition of five groups of mice to understand impact of insulitis, anti–PD-1 treatment, and murine genetic background on the microbiota composition and diversity.

**FIGURE 2.**
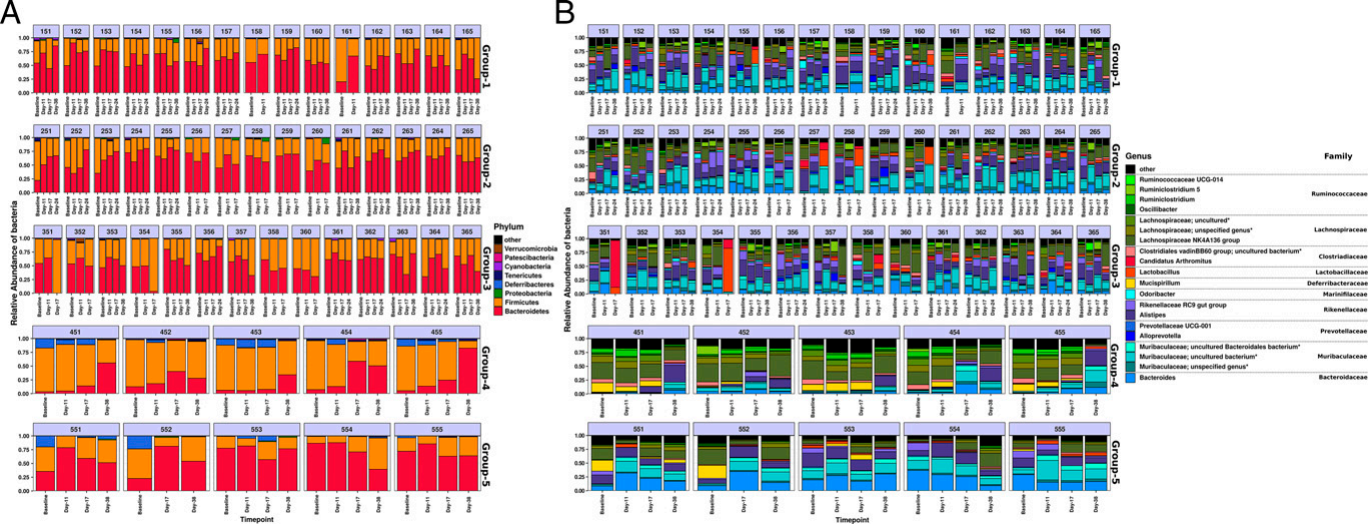
Stacked-bar plot representation showing taxonomic composition of mice gut microbiota across five different groups. (**A**) Phylum- and (**B**) genus-level taxonomic composition of the gut microbiota per mouse and time point for the individual groups. Groups 1–3 are NOD/ShiLtJ (*n* = 15 per group); controls (group 4; *n* = 5) were BALB/c, NOD-SCID (group 5; *n* = 5).

We determined visibly distinct phylum- and genus-level bacterial communities based on genetic background and groups of the mice (Fig. 2). At the phylum level, *Bacteroidetes*, *Firmicutes*, and *Proteobacteria* comprised the most dominant bacterial phyla, whereas *Verrucomicrobia*, *Patescibacteria*, *Cyanobacteria*, *Tenericutes*, and *Deferribacteres* each contributed <1% of total relative abundance (Fig. 2A). Group 1 (treatment naive) and group 2 (isotype IgG treatment) of the NOD/ShiLtJ mouse strain harbored more or less similar *Bacteroidetes* (group 1 = 61.59 ± 14.09% [mean ± SD]; group 2 = 62.13 ± 12.96%) and *Firmicutes* (group 1 = 36.25 ± 14.15%; group 2 = 35.52 ± 12.58%) abundances, whereas group 3 (anti–PD-1 treatment) from the same mouse strain had slightly lower *Bacteroidetes* (51.35 ± 16.55%) and higher *Firmicutes* (43.73 ± 17.01%) levels. Additionally, we found that the relative abundance of *Bacteroidetes* (group 4 = 24.30 ± 22.44%; group 5 = 65.55 ± 18.61%) and *Firmicutes* (group 4 = 67.14 ± 19.59%; group 5 = 29.55 ± 14.01%) were considerably different in the case of the group 4 wild-type BALB/c and group 5 NOD/SCID mouse strain (Fig. 2A). Moreover, we found a considerably higher *Firmicutes*/*Bacteroides* ratio for group 4 mice (2.51 ± 1.33; log_2_ value) followed by group 3 (0.88 ± 0.66; log_2_ value), group 1 (0.71 ± 0.38; log_2_ value), and group 2 (0.69 ± 0.34; log_2_ value). At the genus level, the *Lachnospiraceae NK4A136 group*, *Alistipes, Muribaculaceae uncultured bacterium*, and *Bacteroides* each accounted for an average of >10% of the relative abundance across all groups of samples. We found somewhat comparable abundance of the genus belonging to the families *Lachnospiraceae*, *Muribaculaceae*, *Rikenellaceae*, *Ruminococcaceae*, and *Prevotellaceae* in group 1 (treatment naive), group 2 (isotype IgG treatment), and group 3 (anti–PD-1 treatment) of the NOD/ShiLtJ mouse strain (Fig. 2B). Additionally, we found considerable higher abundance of the genus *Lachnospiraceae NK4A136 group* and *Ruminiclostridium* in the group 4 wild-type BALB/c mouse strain, whereas *Alistipes* and *Bacteroides* were slightly more abundant in the group 5 NOD/SCID mouse strain (Fig. 2B).

**FIGURE 3.**
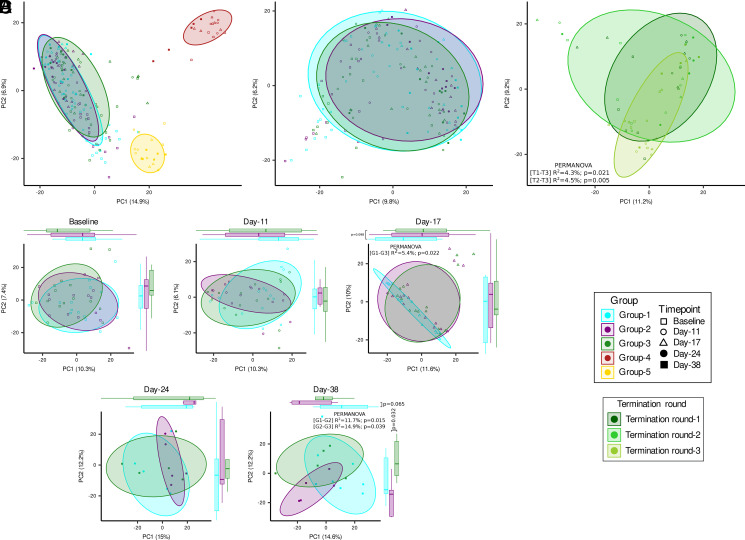
Longitudinal changes in the mice gut microbiota community structure. (**A**) Community composition of all five groups of mice demonstrates the differences between mouse strains: NOD/ShiLtJ (groups 1–3; *n* = 15 per group), BALB/c (group 4; *n* = 5), and NOD/SCID (group 5; *n* = 5). (**B**) Community composition of NOD/ShiLtJ mice from groups 1–3. (**C**) Community composition of NOD/ShiLtJ (groups 1–3; *n* = 15) mice separately at each time point. (**D**) Community composition of NOD/ShiLtJ (group 3; *n* = 5) mice by termination rounds.

Next, we examined how the mouse gut microbiota composition changes in relationship to genetic background, time point, treatment, and termination rounds. We found that genetic background of the mouse accounted for the significant between-sample microbiota variance (PERMANOVA: *R*^2^ = 19%, *p* = 0.0001; Fig. 3A). On comparison of groups 1–3, we found less strong but significant shifts in microbial composition (PERMANOVA: *R*^2^ = 1.8%, *p* = 0.001; Fig. 3B), suggesting microbiota-driven changes associated with anti–PD-1 treatment. Next, we examined longitudinal development of microbiota from NOD/ShiLtJ mice (groups 1–3) separately at each time point (Fig. 3C). As expected, we found no significant microbiota-associated changes at the baseline time point between three groups. However, the microbiota composition differed significantly between group 1 and group 3 mice at day 17, and between group 1 and group 2 and group 2 and group 3 mice at day 38 (posttreatment time points). We subsequently investigated microbiota-level changes with respect to the termination round of animals in group 3 (Fig. 3D). The microbiota community composition shifted significantly between termination rounds 1 and 3 and termination rounds 2 and 3, indicating that animals that were terminated early because of elevated glucose levels had a different microbiota composition from animals that were terminated at later stage.

**FIGURE 4.**
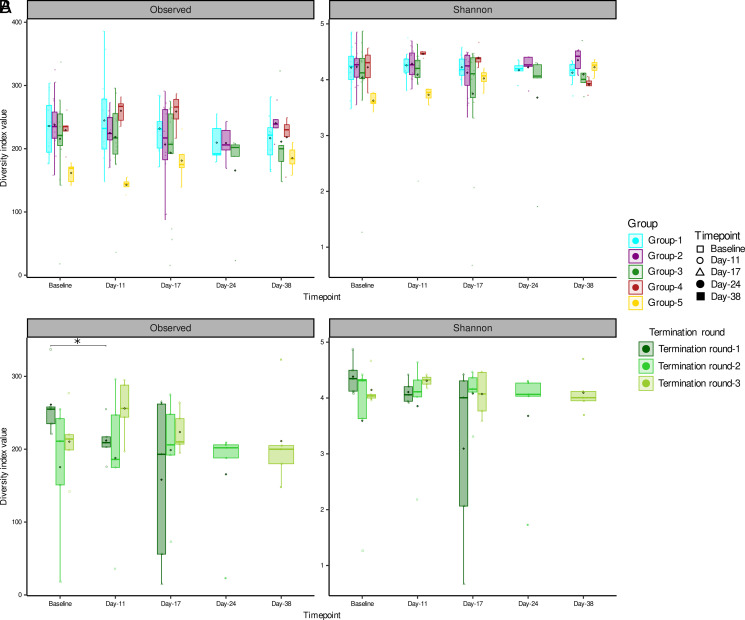
Longitudinal changes in the mice gut microbiota community diversity as measured by observed and Shannon index. (**A**) Diversity of all five groups of mouse strains: NOD/ShiLtJ (groups 1–3; *n* = 15 per group), BALB/c (group 4; *n* = 5), and NOD/SCID (group 5; *n* = 5). (**B**) Diversity of group 3 (NOD/ShiLtJ; *n* = 5) mice by termination rounds. *p < 0.05.

We further measured within-sample α diversity using observed species (community richness estimator) and the Shannon (community richness and evenness estimator) index. We did, however, not find statistically significant changes between any groups at any of the time points compared with baseline samples (Fig. 4A). A comparison of α diversity between termination rounds in group 3 animals showed that species richness on day 11 was significantly lower than at the baseline (Fig. 4B; *p* = 0.05) time point for termination round 1, demonstrating that animals treated with anti–PD-1 have a lower community richness than animals not treated with anti–PD-1. In the case of the Shannon index, we observed the same trend of decreasing diversity for animals from termination round 1 at day 11, but statistical significance (*p* = 0.15) could not be reached.

**FIGURE 5.**
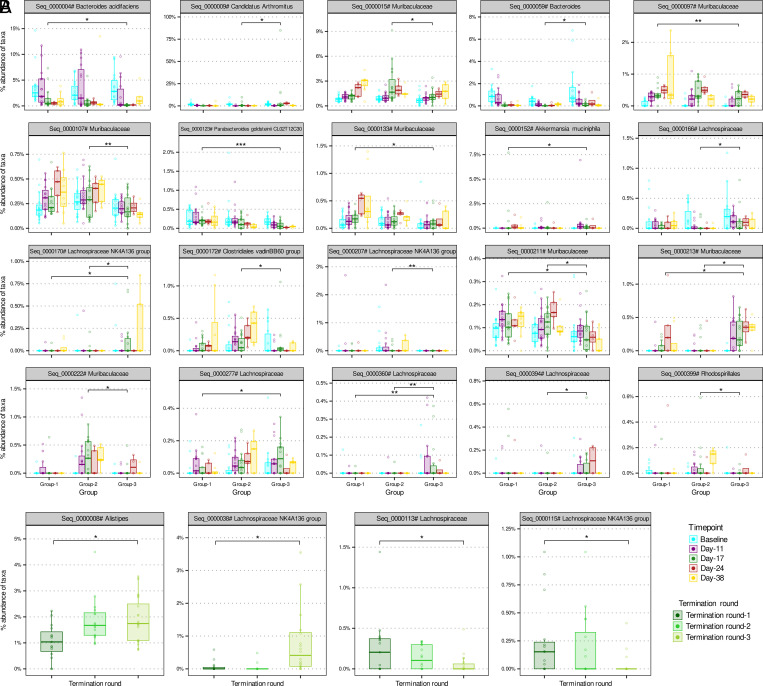
Boxplot representation of significantly different RSVs between groups. (**A**) Groups 1–3 (NOD/ShiLtJ; *n* = 15 per group) and (**B**) termination rounds for group3 (NOD/ShiLtJ; *n* = 5 per termination round). Significant *q* values (FDR) are noted as follows: *q < 0.1, **q < 0.01, ***q < 0.001.

We next identified 20 RSVs that were significantly different (*q* value < 0.1) between animals from groups 1–3 (NOD/ShiLtJ; Fig. 5A). Particularly, we identified significantly reduced (*q* value = 0.013) abundance of the RSV belonging to *Bacteroides acidifaciens* (Seq_0000004) in group 3 (anti–PD-1) compared with group 1 and group 2, which has been previously reported to improve glucose intolerance and insulin resistance. Four different RSVs belonging to *Lachnospiraceae* (Seq_0000166, Seq_0000277, Seq_0000360, Seq_0000394), previously associated with T1D pathogenesis (37), were found to be significantly increased (*q* value < 0.1) in group 3 compared with group 1 and group 2. Four different RSVs from the *Muribaculaceae* family of the phylum Bacteroidetes (Seq_0000015, Seq_0000097, Seq_0000133, Seq_0000211) were significantly decreased in group 3 compared with group 1. *Parabacteroides goldsteinii* has been associated with reduced gut inflammation and insulin resistance in type 2 diabetes (38), and the RSV from this species (Seq_0000123) was found to be significantly decreased in group 3 compared with group 1. In addition to that, we found a significant decreased in abundance of RSV belonging to *Akkermansia muciniphila* (Seq_0000152) in group 1 compared with group 3, suggesting its association with anti–PD-1 responses. Statistical comparison of RSV abundance between termination rounds in group 3 animals revealed three different *Lachnospiraceae* RSVs (Seq_0000038, Seq_0000113, Seq_0000115) and one *Alistipes* RSV (Seq_0000008) as significantly (*q* value < 0.1) different in termination round 1 than round 3 (Fig. 5B). Apart from that, we even found significantly (*q* value = 0.079) higher abundance of the phylum *Proteobacteria* in termination round 1 compared with round 3 (data not shown). Pathway analysis using imputed metagenomics based on 16S rRNA sequences revealed tryptophan metabolism as significantly decreased (*q* value = 0.045) in group 3 compared with group 1 (Supplemental Fig. 3), which has also been described in T1D patients and mouse model (39).

Finally, we correlated glucose levels with abundance of microbial taxa obtained over time using repeated measure correlation analysis. Supplemental Fig. 4A shows significantly associated (*q* value < 0.1) taxa with glucose level in animals from groups 1–3. At the phylum level, we found significant positive correlation (*r *= 0.38, *q* value = 0.057) between abundance of *Proteobacteria* and high glucose levels in group 3; however, this was not the case for group 1 (*r* = 0.12, *q* value = 0.85) and group 2 (*r* = 0.07, *q* value = 0.82). On correlation of abundance of RSVs with glucose level measurements, we found a clear trend of negative association of *B. acidifaciens* (*r* = −0.44, *q* value = 0.14) and *P. goldsteinii* (*r* = −0.41, *q* value = 0.14) in the group 3 animals with elevated glucose level. When correlating the abundance of RSVs with glucose level in group 3 animals according to their termination rounds, we found a clear trend of negative association of RSVs belonging to *B. acidifaciens* and *P. goldsteinii* and a clear trend of positive association of the *A. muciniphila* RSV in termination round 1 (Supplemental Fig. 4B).

## Discussion

Our study confirms an accelerated onset of T1D, increased GAD-reactive T cell frequency in spleen, precipitated destruction of β cells, triggering of high glucose levels, and pancreatic islet reduction in susceptible NOD/ShiLtJ mice treated with murine anti–PD-1 mAb. The commensal gut microbiota regulates the homeostasis and maturation of immune cells in the intestinal lamina propria and in the periphery. Mammals have coevolved with a specific consortium of gut bacteria capable of mounting proinflammatory and anti-inflammatory immune responses (40). Segmented filamentous bacteria are powerful inducers of Th17 and Th1 cells whereas *Clostridia *species and *Bacteroides fragilis* are regulatory T cell stimulators (41–44). Around 70% of immune cells are located in the intestine and, if in circulation, they can enter the gut via gut homing markers, such as α_4_β_7_ integrin. Given the central role of effector T cells in autoimmunity, it has long been suspected that alterations in the composition and function of the gut microbiota, particularly during the neonatal period, might affect onset and severity of autoimmunity via the priming of effector T cells in the gut (45). Changes in the microbiota in human T1D and NOD mice are well studied; common findings include increased abundances of *Bacteroides* species and deficiency of bacteria that produce short-chain fatty acids (46, 47). In addition, increased intestinal permeability and decreased microbial diversity after islet autoimmunity but before T1D diagnosis has been reported (48). NOD mice fed with a specialized diet resulting in high bacterial release of short-chain fatty acids (acetate and butyrate) were almost completely protected from T1D (49). As we overlay genetics in T1D, the microbiome in diabetes with anti–PD-1 treatment and age-dependent effects on the microbiome, the first strong observation was the confirmation of common outcomes in T1D, such as reduced diversity and an increase of abundance of *Bacteroidetes* as well as a decrease in *Firmicutes* and *Tenericutes*. Interestingly, decreased abundance of *B. acidifaciens* and *P. goldsteinii* in group 3 indicates a contribution to an accelerated diabetes onset driven by anti–PD-1 treatment. Several reports associated both species with enhanced intestinal integrity, reduced levels of inflammation and improved insulin sensitivity in type 2 diabetes when mice were orally treated with living bacterial strains (38, 50). The decreased abundance of these two bacterial species at day 11 indicates a prodromal role of the microbiome in the onset of T1D.

We found higher abundance of *Proteobacteria* in termination round 1 (high glucose levels), which fits to an inflammatory status characterized by aerobic conditions known for diabetes. We also identified elevated *Lachnospiraceae* abundances in termination round 1, further confirming a shift of composition in an accelerated fashion compared with termination round 3 (low glucose levels) because it has been reported that Lachnospiraceae actively impairs glucose metabolism, leading to inflammation and promoting the onset of T1D (37). The decreased abundance of the genus *Alistipes* in termination round 1 compared with termination round 3 has not yet been described in NOD mice. However, *Alistipes* species have been linked to improved responses to anti–PD-1/PD-L1 therapies in tumor mouse models and cancer patients (51), pointing to proinflammatory properties via activation of innate and adaptive immune cells. Those discrepancies might be due to differences in mouse models and methodologies applied in this and other studies, which will require further microbiome investigations, for instance complemented by metagenomics and metatranscriptomics sequencing for improved taxonomic potential and insights into potential and actual function. A limitation of our study is the small number of animals that were included, and it is possible that identified significant taxa are not completely correlated with findings in other studies on the role of anti–PD-1 treatment on microbiota changes.

Along those lines, checkpoint inhibitors play a pivotal role in the treatment of various advanced-stage cancers, as evidenced by improved immune responses with PD-1/PD-L1 and CTLA-4 therapies (52). Importantly, life-threatening side effects are frequently observed, such as cytokine release and immune-related adverse effects, including colitis, hepatitis, and diabetes. It is therefore important to accurately identify the subset of patients who benefit or suffer the most from checkpoint blockades. We found that *A. muciniphila* abundances increased in the anti–PD-1–treated group compared with naive NOD mice. Additionally, this species has been associated with responders in anti–PD-1 therapies where CD8 T cells were increased in the tumor and gut tissue when fecal microbiota transplantation was performed from human cancer patients into germ-free mice (16). Members of the *Akkermansia* genus are mucus degraders and are able to directly stimulate the intestinal epithelium and innate immune cells of the intestinal lamina propria. The increased abundance of this species confirms previous results in cancer mouse models and cancer patients; importantly, our results emphasize that this bacterial species is associated with accelerated diabetes in the NOD mice, as naive and isotype-treated NOD mice did not show a significant increase. Moreover, we observed a trend toward positive correlation of high glucose levels and increased abundance of *A. muciniphila* in group 3, which points to an association of this mucin-degrading species with an accelerated or exacerbated autoimmune outcome after anti–PD-1 treatment. Further investigations are, however, necessary to fully understand whether the mucus layer changed, or gut permeability increased, due to the onset of diabetes and that *A. muciniphila* abundance increased because of the lack of barrier function. Finally, it is tempting to ask whether patients before and during therapy with checkpoint inhibitors should be monitored for their abundance of these three species to identify and stratify patients who have a high risk for developing diabetes. The prodromal role of bacterial species in T1D that is described in the current study provides the opportunity of adjusting dosage and subsequent safety in a personalized fashion when implemented in the clinic. Also, it is worth considering how to harness the prodromal role of other bacterial species in other mouse models of autoimmunity, such as in the experimental autoimmune encephalomyelitis model, to investigate potential early microbial markers for the risk of inflammation of the nervous system.

In summary, our study shows that anti–PD-1 treatment has the potential to accelerate the onset and severity of T1D in susceptible NOD mice. Besides the observation of immune cell infiltrates, GAD-reactive T cells, and high glucose levels, we describe, to our knowledge for the first time, longitudinal changes in the gut microbiota following anti–PD-1 treatment by identifying bacterial species as early markers before T1D onset. These results will pave the way for further investigations to enable the identification and stratification of susceptible patients who are at risk for immune-mediated adverse events or even autoimmunity in CIT based on early microbial markers. As such, our approach provides guidance for treatment regimens potentially improving clinical outcomes.

## Supplementary Material

Supplemental Figures 1 (PDF)
