# Supplementary material for: Gut Microbiota Is Associated with Onset and Severity of Type 1 Diabetes in Nonobese Diabetic Mice Treated with Anti–PD-1
Source: Immunohorizons. 2023 Dec 26;7(12):872–85. doi: 10.4049/immunohorizons.2300103 (PMC10759162; doi:10.4049/immunohorizons.2300103)
Supplement: Supplemental Figures 1 (PDF) [file IH_2300103_Supplemental_1.pdf]

Supplement Figures

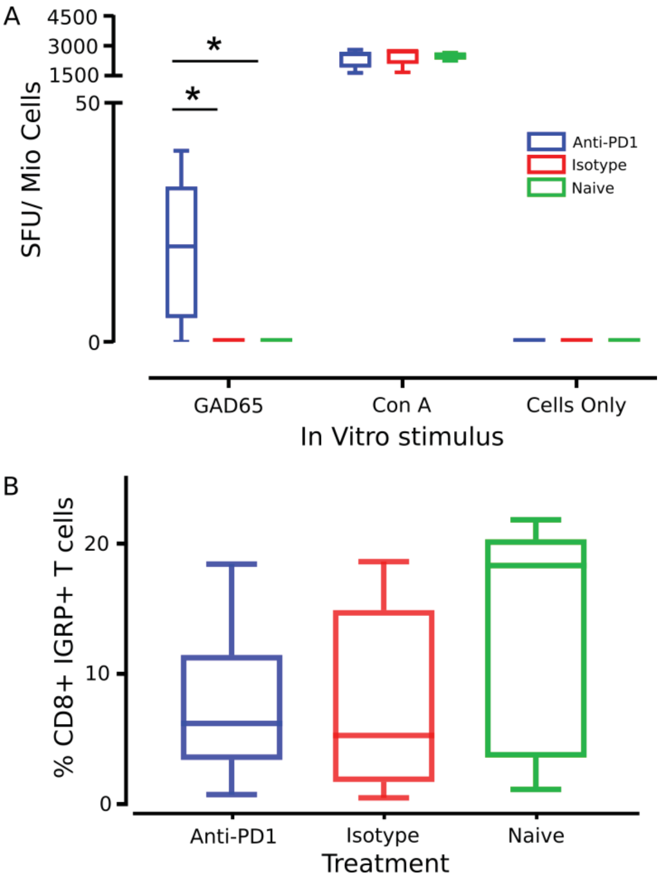

Supplementary Figure 1: Immunological assessments. Immunological analysis displayed increased GAD65-specific but not insulin-specific autoreactive T cells in NOD/ShiLtJ mice. (A) IFNg production after in vitro stimulation of splenocytes with GAD65 (10µg/ml), Concanavalin A (5µg/ml) or non-treated cells applying ELISPOT (n= 15). (B) Percentage of IFNg producing CD8+ T cells in the spleen and draining lymph nodes of NOD/ShiLtJ as determined by a tetramer staining (insulin: MHC complex). Statistical analysis was performed by using t test (* indicates $p < 0.05$).

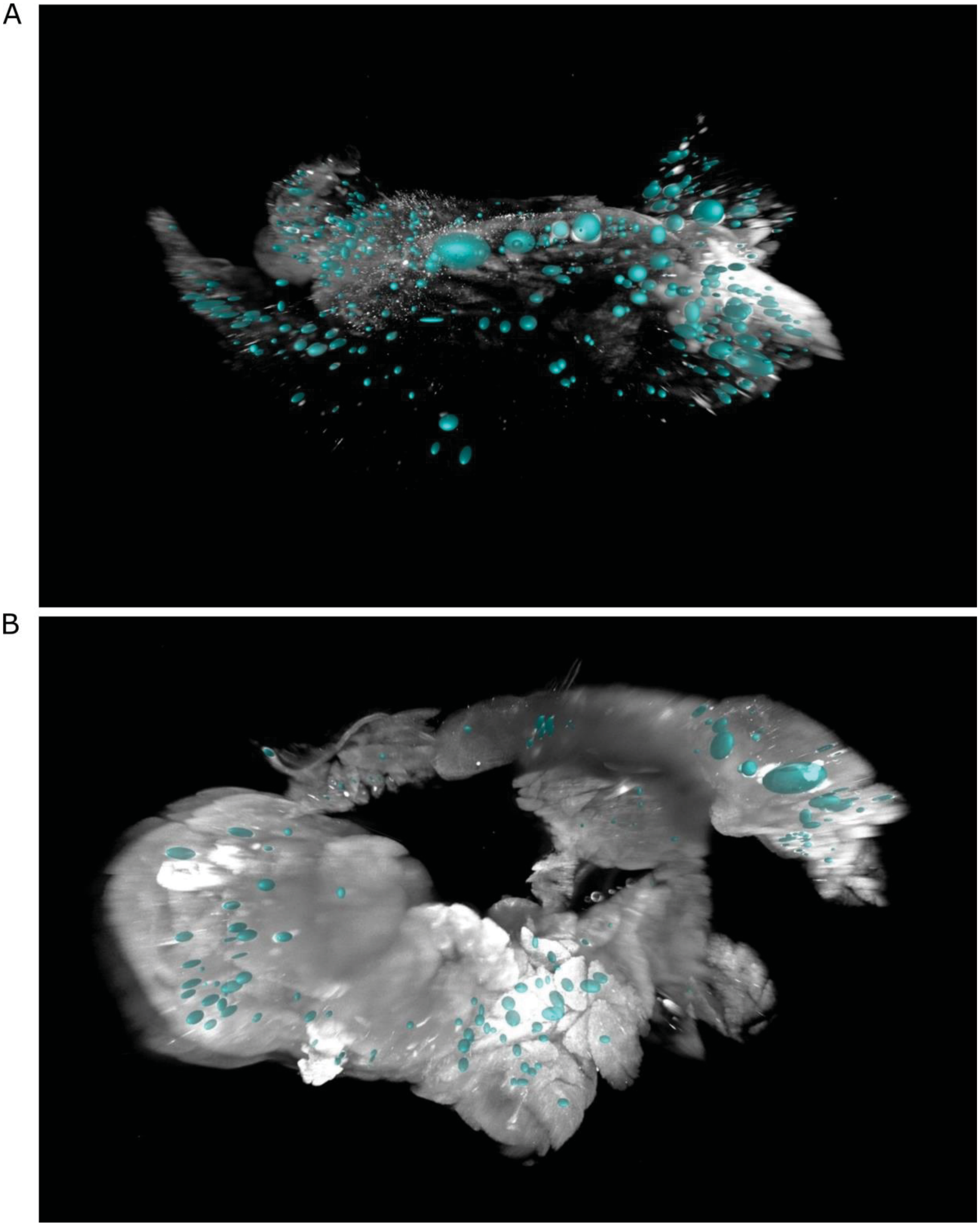

Supplementary Figure 2: 3D reconstruction of the whole pancreas with Lightsheet microscopy. One animal from the (A) control (no. 151) and (B) anti-PD-1 treated (no. 362) group were processed respectively. Due to the experimental design, there is no statistical evaluation possible. This is only for illustration of the heterogeneity of islet distribution per animal, which indicates a potential for a decrease of islets in the anti-PD-1 treated animal. Further analysis on more samples is needed to draw the right conclusions.

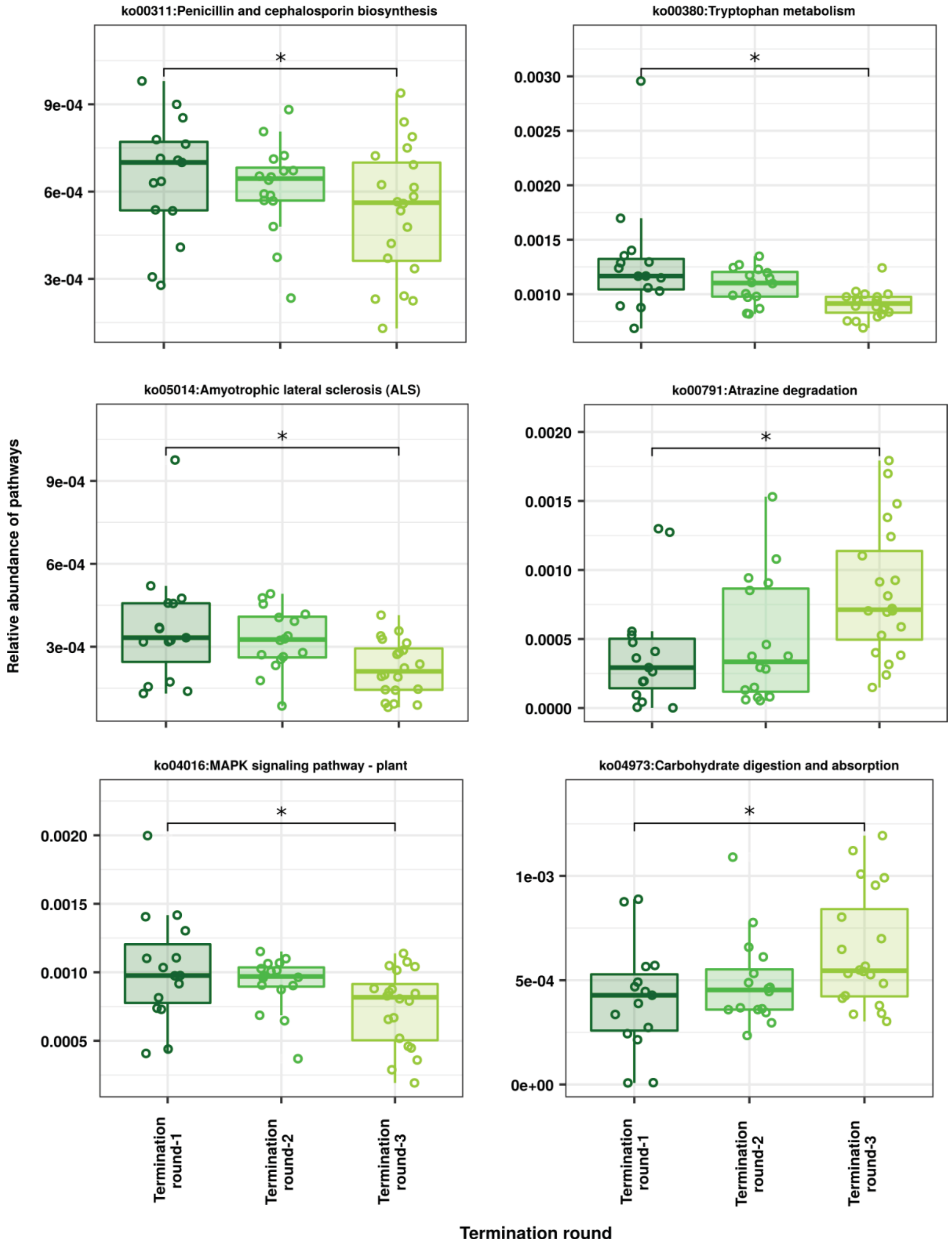

Supplementary Figure 3: Significantly different KEGG pathway identified based on imputed metagenomics using Piphillin. Pathways significantly different by termination rounds in group-3 (NOD/ShiLtJ). Significant q-value (FDR) marked as asterisk. * = q < 0.05

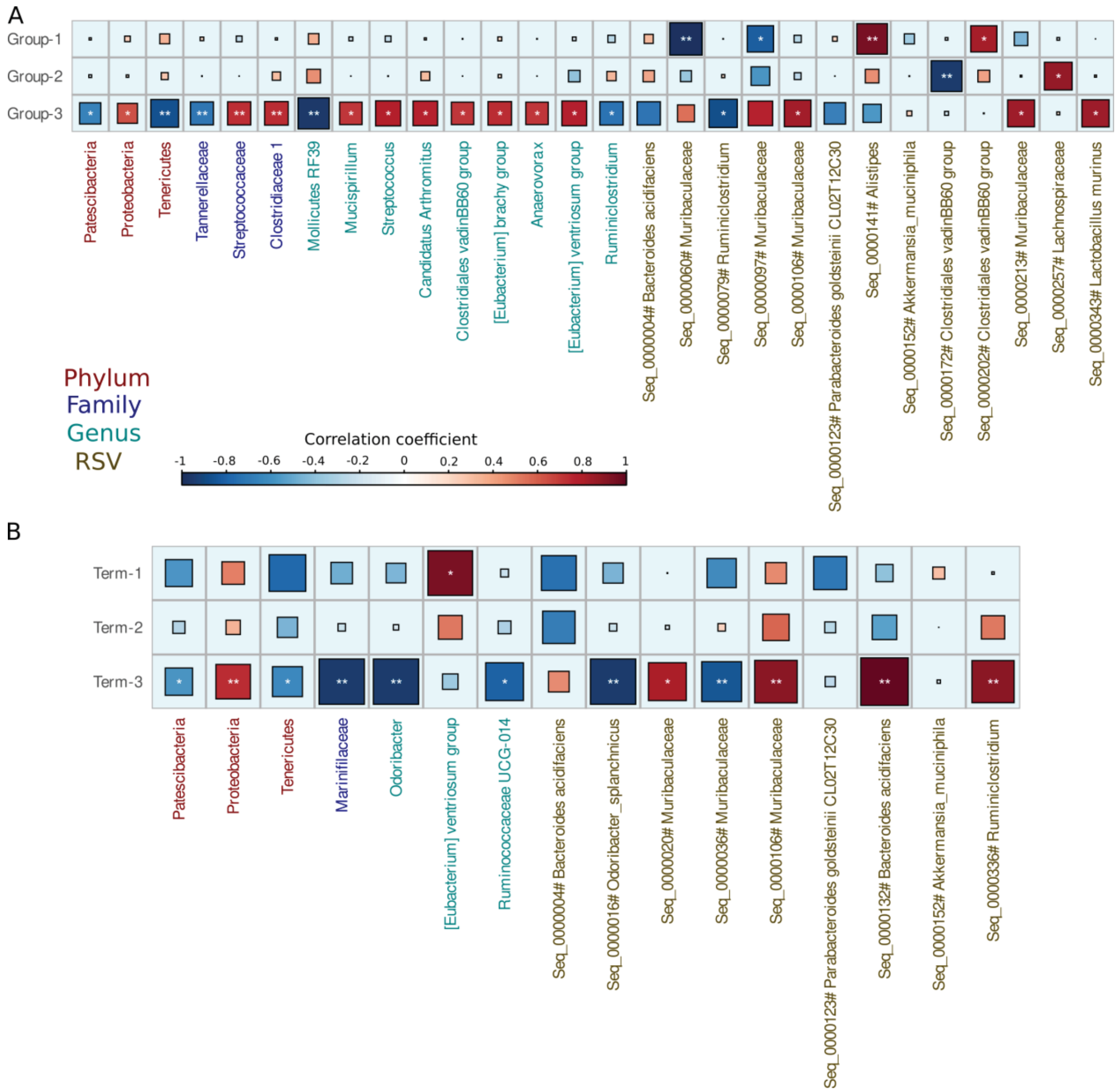

Supplementary Figure 4: Taxa displaying significant association with glucose level in (A) groups 1-3 (NOD/ShiLtJ) and (B) termination rounds 1-3. Significant q-value (FDR) marked as asterisk. * = $q < 0.1$, ** = $q < 0.01$, *** = $q < 0.001$.
